# Change in physical activity is not associated with change in mental distress among adolescents: the Tromsø study: Fit Futures

**DOI:** 10.1186/s12889-019-7271-6

**Published:** 2019-07-09

**Authors:** Ida Marie Opdal, Bente Morseth, Bjørn Helge Handegård, Kjersti Lillevoll, Helga Ask, Christopher Sivert Nielsen, Alexander Horsch, Anne-Sofie Furberg, Simon Rosenbaum, Kamilla Rognmo

**Affiliations:** 1 Department of Psychology, UiT The Arctic University of Norway, Tromsø, Norway; 2 School of Sports Sciences, UiT The Arctic University of Norway, Tromsø, Norway; 3 Regional Centre for Child and Youth Mental Health and Child Welfare, UiT The Arctic University of Norway, Tromsø, Norway; 4 Norwegian Institute of Public Health, Oslo, Norway; 5 Department of Computer Sciences, UiT The Arctic University of Norway, Tromsø, Norway; 6 Department of Community Medicine, UiT The Arctic University of Norway, Tromsø, Norway; 7 School of psychiatry, University of New South Wales, Sydney, Australia; 8 Black Dog Institute, Sydney, Australia

**Keywords:** Adolescence, Youth, Physical activity, Mental distress, Depression, Anxiety, Fit futures, Accelerometry

## Abstract

**Background:**

Previous research shows that physical activity has a protective effect on mental distress in adults, but the relationship is less researched and seems more ambiguous for adolescents. Studies in this field have typically been cross-sectional by design and based on self-reported physical activity measures, which are known to be vulnerable to response bias. The aim of this study was to investigate the relationship between change in objectively assessed physical activity as measured by accelerometer and change in mental distress among adolescents using longitudinal data from The Tromsø Study: Fit Futures.

**Method:**

This study was based on data from 676 upper-secondary school students (mean age 16.23 years at baseline, 45.26% boys) from The Tromsø Study: Fit Futures. Physical activity, mental distress and covariates were measured at baseline (T1) and follow-up (T2) 2 years later. Physical activity was objectively measured with an ActiGraph GT3X accelerometer over 7 days. Mental distress was measured with the Hopkins Symptom Checklist-10 (HSCL-10). Change score variables were computed as the difference between T1 and T2 in number of steps, number of minutes of moderate to vigorous physical activity (MVPA) and mental distress between T1 and T2, and analyzed using linear regression analysis.

**Results:**

Changes in steps per day were not associated with changes in mental distress in neither the crude, partially, nor fully adjusted model. Neither was changes in minutes of MVPA per day. Interaction effects between change in both steps per day and minutes of MVPA and gender were also not statistically significant, nor was the interaction effects between baseline levels of mental distress and physical activity.

**Conclusion:**

The results of our study indicate that for adolescents in the sample, change in physical activity is unrelated to change in mental distress over a two-year period.

More than one in ten children and adolescents suffer from a mental disorder [1]. As the median age of onset for experiencing a mental disorder is during the teenage years [2], adolescence may be a crucial period for preventing the development of mental health problems.

Physical activity seems to be related to mental health problems, such as anxiety and depression [3]. The relationship may be explained by low levels of physical activity among individuals with high levels of mental distress, whereas another possible explanation is that physical activity elicits physiological responses that enhance mood, such as higher levels of norepinephrine, endorphins and serotonin, and lower hormonal responses from stress [4, 5]. High intensity and frequent physical activity of young athletes compared to non-athletes has been shown to be associated with lower anxiety and fewer depressive symptoms, and may serve as an indication that some of the effect of physical activity on mental distress is explained by physiological factors associated with increased hormonal responses at higher levels of intensity of exercise [6–9]. A psychological explanation for the relationship may be that physical activity causes feelings of mastery and control [4], influences self-esteem [10], body composition [11], acts as a distractor from stressors, negative thoughts and rumination [4], and may provide an arena for the individual to learn social skills [12] and get a social network [4, 13]. For example, single bouts of exercise were found to impact positively on mood and rumination among other factors in inpatients with mental disorders [14]. The context in which activity is performed may also be of importance, as participation in organized sports has been found to be associated with lower odds of depressive symptoms [15].

The relationship between physical activity and mental health among adults is well established, however, less studies have investigated the relationship among adolescents. The majority of the studies in the field are cross-sectional [16], with the overall result indicating that higher levels of physical activity are related to lower levels of depressive symptoms [17]. However, the strength of the relationship is small to moderate, and the validity and reliability of the results are limited by methodological weaknesses relating to measurement and study design as a majority of the studies has relied on single-item self-report measures of physical activity [17]. In addition, results from cross-sectional studies are not informative regarding changes in the relationship over time. In general, physical activity levels seem to decrease over time during adolescence [18], whereas the prevalence of mental disorders increases [19]. It also seems that recent cohorts of girls experience an increase in internalizing symptoms compared with older cohorts [20], indicating a change in the cohorts, making interpretation from cross-sectional studies difficult. Longitudinal studies are therefore required to investigate if change in physical activity is associated with change in mental distress.

The results of existing longitudinal studies on the relationship seem to depend upon the type of measurement of physical activity. Longitudinal studies using self-report measures tend to find a relationship between physical activity and mental health problems over time [4, 21–24], although not consistently [25, 26]. However, the few studies using objective measurements of physical activity, e.g. step counter or accelerometer, have not found a significant relationship [27, 28]. Objectively measured physical activity and self-reported physical activity are not equivalent, as accelerometers accurately measures the movement in physical activity behaviors and self-reported measurements often aim at measuring the normal level of physical activity in reported time-periods [29]. Whilst self-reported data is based on subjective opinion and recall of past experiences, objective measured data collect the overall total physical activity during a specified time period. The use of self-reported physical activity is subject to recall and response bias [30], and research show that adolescents tend to over-report time spent in moderate and vigorous physical activity compared to objective accelerometer data [31]. Whilst the results of the longitudinal studies using self-report data tend to show significant effects of physical activity, a recent meta-analysis shows that the overall effect of physical activity on depressive symptoms is small [32].

To the best of our knowledge, only two longitudinal studies have used objective measures of physical activity to investigate the association between physical activity and mental health problems among adolescents. Van Dijk et al. [28] found that change in objectively measured physical activity was not associated with change in mental health among 158 adolescents aged 13 years over a one-year period. Overall, physical activity declined by 15.3%, and depressive symptoms by 12.1% between baseline and one-year follow up. Toseeb et al. [27] also found no longitudinal association between objectively measured physical activity at baseline and the development of symptoms of depression during a three-year period for 736 adolescents in the UK aged 14.5 years at baseline. Data on change in physical activity was not available from the Toseeb et al. study, however they found a small decrease in depressive symptoms (5.7% decrease in total score) and an increase in major depression diagnoses by 57% between baseline and the three-year follow up study.

As the measurement method seems to impact the results of the studies investigating the longitudinal relationship between physical activity and mental health outcomes, it is imperative that more studies using objective measurements are performed, in order to increase the knowledge regarding this relationship and shed light upon the apparent discrepancy in results of studies using different methodology. The current study aims to investigate the degree to which change in objectively measured physical activity is related to change in mental distress, operationalized as change in symptoms of anxiety and depression, among adolescents, by using data from The Tromsø study: Fit Futures. As the studies based on objective measurements report different findings than the studies based on self-report, hypothesizing regarding the results of the present study is difficult. However, as most previous studies have shown a relationship over time, and as the biological and psychological explanatory models provide a solid foundation for the mechanisms involved, we hypothesize that change in physical activity is inversely related to change in mental distress over a two-year period. We also hypothesize that the participants with the highest levels of mental distress at baseline benefit more from a change in physical activity between baseline and follow-up than the less mentally distressed individuals, and that higher levels of change in physical activity over time are related to greater change in mental distress.

## Method

### Study design and sample

In 2010–11, all first-level upper secondary school students in two municipalities in the county of Troms in Northern Norway were invited to participate in the general health study The Tromsø study: Fit Futures 1 (T1), described previously [33]. The study was conducted during school hours, and participants were transported to the research site at the Clinical Research Unit, University Hospital of North Norway, Tromsø.

In total, 1,117 students were invited to participate in T1, out of which 1,038 (92.9%) agreed. In 2012–13, all attendees in T1 and all students at the third level of the same upper secondary schools as in T1, were invited to participate in The Tromsø study: Fit Futures 2 (T2). In total, 1,129 students were invited to T2, out of which 870 (77%) agreed. 694 (67%) students who participated in T1 also participated in T2, and 676 of these are included in the current study after multiple imputation. The majority of the participating sample was 15–17 years old in T1 and 17–19 years old in T2.

Both T1 and T2 included a web-based questionnaire, a clinical examination and interview performed by trained research nurses at the study site. The participants filled in a web-based general questionnaire (Questback) about family, lifestyle, health and disease, and received an ActiGraph GT3X accelerometer and wearing instructions. Height and weight were measured according to standardized procedures. Information about diagnosis of any chronic disease was obtained from the interview. All participants provided written informed consent; participants younger than age 16 also provided written informed consent from a guardian.

### Measurements

#### Physical activity

Physical activity was measured using the ActiGraph GT3X accelerometer. The validity of the ActiGraph GT3X has been well established and is superior to self-reported physical activity as assessed by self-report questionnaire [30, 34], although it tends to underestimate cycling and unstructured activity [31]. The subjects were instructed to wear the ActiGraph on their dominant hip for 7 days, and to only take it off when sleeping, showering or swimming. Participants with at least 10 h wear-time for at least four out of the 7 days were considered to have valid data due to the possible variation in physical activity in the period of measurement.

ActiLife software provided by the manufacturer (ActiGraph, LLC, Pensacola, USA) was used for ActiGraph device initialization and data downloaded as 10-s time intervals (epochs). The further data processing was done with the Quality Control & Analysis Tool (QCAT). The data was aggregated to epochs of 60 s duration for the analysis. This was considered reasonable for the basic variables related to volume, intensity and duration of physical activity used in this study. For wear-time calculation we used the triaxial algorithm described by Hecht et al. [35] as it conforms to previous research definition of non-wear time [36].

ActiGraph variables of interest in this study are number of steps on 10-s time intervals (epochs) and minutes in moderate to vigorous physical activity (MVPA) per valid day, based on uniaxial cut-points by Freedson et al. [37]. Change score variables for steps (steps per valid day at T2 minus steps per valid day at T1) and MVPA (minutes MVPA per valid day at T2 minus minutes MVPA per valid day at T1) were calculated and served as predictor variables in the analyses. Change score for steps per valid day estimates the overall physical activity level, while minutes in MVPA per valid day was included to investigate the impact of change in intensity of physical activity over time in change in mental distress. To make it easier to visualize the change, the total number of steps per valid day was divided by 1000. In order to see if the degree of change from T1 to T2 was related to change in mental distress, steps per day was categorized into four groups, a high negative change (more than 3000 steps reduction at T2), a small negative change (0 to 3000 reduction in steps at T2), a small positive change (0–3000 steps increase at T2), and a high positive change (more than 3000 steps increase at T2). This variable was used in supplemental, interaction analyses.

#### Mental distress

Mental distress was measured by the Hopkins Symptom Checklist-10 (HSCL-10) in the general questionnaire. HSCL-10 is a well-validated instrument measuring symptoms of anxiety (4 items) and depression (6 items) during the previous 7 days [38]. Response categories were “none” (1), “slightly”, “much” and “very much” (4). The average score of the 10 items was calculated for T1 and T2, and a difference score variable (T2 mental distress minus T1 mental distress) was created and served as the outcome variable in the analyses. In addition, interaction analyses were conducted in order to examine if the relationship between change in physical activity and change in mental distress is dependent upon mean baseline levels of mental distress.

#### Covariates

A number of variables may impact change in physical activity and change in mental distress, and accordingly we have included demographic variables (e.g. socioeconomic status [39], sex and age [40]), social level variables (e.g. social network [41]), and health or lifestyle related variables (e.g. smoking [42], chronic pain [43], BMI [44] and sleep [45, 46]) as covariates in the analyses. Change in variables that were measured at both T1 and T2 were included as covariates, in addition to baseline levels of the same variables.

##### Demography

Sex (female = 0, male = 1) was included as a possible confounder in the analyses. At T1, the respondents were asked to indicate the highest level of completed education of their mother and father, which served as a proxy for socioeconomic status (high/low, with low including education up to and including secondary vocational education) in the analyses. As season of the year might significantly impact the level of physical activity, especially in regions north of the Arctic Circle, a seasonal variable indicating the time of ActiGraph measurement at T1 and T2 was included in the analyses to control for measurements during summertime (May to June and September to October, value = 0), or wintertime (November to April, value = 1).

##### Social network and school motivation

Considered quitting or taking a break from school in T1 (yes/no) was included as a possible confounder. Social network was measured at T1 and T2 by five items asking about the degree to which the respondents find it hard to make friends, have many friends, feel accepted among his/her peers, feel liked among peers, and feel popular among peers. Change in social network between T1 to T2 was calculated (T2-T1) and used as a covariate in the analyses, as was baseline social network.

##### Health and lifestyle related variables

In the interview in T1, the respondents were asked if they had any chronic or persistent diseases, and if they reported that they did, they were asked to specify up to five diagnoses. A dichotomous variable was created and coded as follows: 0 = no chronic disease present, 1 = one or more chronic diseases present. The respondents also reported if they suffered from chronic pain (yes/no). Weight and height were measured at the examination site and BMI was calculated by dividing the participants weight by their height squared. In order to take into account body growth, BMI at baseline was categorized in age and sex specific categories with cut-offs defined by previous research [47], and used as a covariate in the analyses. Baseline levels and change in self-reported health between T1 and T2, measured by a five-point Likert type scale, were also included as covariates. Furthermore, smoking was assessed in the questionnaire at T1 (“no”, “sometimes”, “daily”), and was dichotomized into no and sometimes/daily.

In the general questionnaire in T1, sleep delay was measured by asking the respondents to indicate how long they normally lie awake before falling asleep (sleep onset latency) on weekdays and weekends. Response categories ranged from “30 minutes or less” to “three hours or more”. A sleep onset latency over 30 min on more than 3 days per week is considered a clinical marker for insomnia [48]; consequently, a dichotomous variable was created, with a cut-off at spending more than 30 min falling asleep. The respondents were also asked about how many hours of sleep they normally get per night. Response categories ranged from “four hours or less” to “12 hours or more”. At last, the degree to which the respondent felt like they get enough sleep was measured, and the response categories were “yes, absolutely enough”, “yes, normally enough”, “no, somewhat insufficient”, “no, clearly insufficient” and “no, far from sufficient”. The responses were dichotomized into “yes” (the two first response categories) and “no” (the three last response categories).

Questions asking about the number of times a week the participants consumed unhealthy food such as pizza, hamburger or hot dogs, chocolate or sweets, snacks and soft drinks with sugar, or healthy food such as fat fish, lean fish, fruit, vegetables, cod liver oil and vitamin or mineral supplements were included in the questionnaires at T1 and T2. The response categories ranged from “rarely/never” to “every day”. Two variables were created – one average score variable based on the four unhealthy diet items and one average score variable based on the six healthy diet items. Change scores between unhealthy and healthy diet at T1 and T2 were calculated and served as covariates in the analyses, in addition to unhealthy and healthy diet at T1.

### Treatment of missing data

In order to increase power to detect an effect, as well as reduce possible bias due to missing values, Multiple imputation (IBM SPSS 25 for windows) was used to impute T1 and T2 ActiGraph data and parental education. It was set a requirement prior to the imputations that only missing ActiGraph and socioeconomic data of participants who had questionnaire data from both T1 and T2 were to be imputed due to their representation of a high proportion of the missing data. Participants who had missing data were not significantly different from those with complete data, and the observed values were perceived as good predictors for the multiple imputation. At T1, 608 (58.6% of total number of participants) had valid ActiGraph data, whereas 420 (48.3% of total number of participants) had valid ActiGraph data at T2. A predictive model consisting of all variables included in the analyses, including T1 and T2 steps per valid day and minutes in MVPA per valid day, was used to create 50 imputed datasets that subsequently were pooled and analyzed. After imputations, 35.2% of the data on steps per day, 35.1% of MVPA, 25% of socioeconomic status of mother and 29.3% of socioeconomic status of father were imputed. After imputation, 676 participants had complete data on all the variables used in the analyses.

### Data analysis

This study investigates the assumption that the difference between T1 and T2 levels of the dependent variable is due to change in levels of physical activity, and thus, change scores between T1 and T2 were calculated for the predictor variables steps per valid day and minutes in MVPA per valid day, and the outcome variable mental distress. Analysis on change scores gives unique information on the relationship between change in the independent and dependent variable over two time points, and is preferred over other longitudinal methods in this study due to the specific research question that we wish to address and due to the robustness of multiple imputation in estimating missing values. Two sets of stepwise linear regression analyses were conducted, using the statistical software SPSS version 25.0 (IBM Corporation, Armonk NY, USA) for Windows® [49]. In the first model, change in steps was the predictor of change in mental distress. In the second model, change in minutes in MVPA was the predictor of change in mental distress. The analyses were run both unadjusted and adjusted by several potentially influential confounders. In the first block of the analyses, change in steps was entered alone, in order to estimate the crude effect. Next, in block two, the demographic variables were entered: sex, age, socioeconomic status of the mother and father, season of actigraphy measurement. In block three, the social level variables social network (change between T1 and T2 and baseline levels) and having considered dropping out of school were entered. Finally, health and lifestyle related variables were included in block four, including chronic disease, chronic pain, sleep onset latency, hours of sleep, sufficient amounts of sleep, baseline BMI, self-reported health (change between T1 and T2 and baseline levels), healthy and unhealthy diet (change between T1 and T2 and baseline levels) and smoking. Interaction effects of change in physical activity and sex, as well as change in physical activity and baseline levels of mental distress were investigated in the fully adjusted model in separate analyses. In addition, an analysis of covariance was performed in order to see if a high negative change in steps per day from T1 to T2 was related to change in mental distress, compared to a high positive change adjusted for sex, age, socioeconomic status, BMI, season, and sleep onset latency.

The analyses were also run on complete cases. Since there were no differences in the results, only results from the analyses based on multiply imputed data are reported.

## Results

### Descriptive statistics

Descriptive statistics are shown in Table 1 and Table 2. Most of the participant’s physical activity was tested during wintertime, and a majority of the participants reported that their mother and father had low levels of education (see Table 1). Mean levels of steps per day and MVPA were also relatively stable from T1 to T2, although somewhat lower at T2 than at T1. The mean levels of MVPA at T1 and T2 were lower than the recommended 60 min of MVPA per day [50].

**Table 1:** Frequencies and percentages of T1 subject characteristics. The Tromsø Study – Fit Futures

	All (*N* = 676)
Sex	
Boys	306 (45.3%)
Chronic disease T1	
No	474 (70.1%)
Yes, one or more	202 (29.9%)
Chronic pain T1	
No	512 (75.7%)
Yes	164 (24.3%)
SES mother T1	
Low	391.1 (57.8%)
High	284.9 (42.2%)
SES father T1	
Low	433.7 (64.2%)
High	242.3 (35.8%)
Feeling of enough sleep T1	
No	389 (57.5%)
Yes	287 (42.5%)
Sleep onset latency (30 min) T1	
No	429 (63.4%)
Yes	247 (36.6%)
Using the ActiGraph in the winter	
T1	552 (81.6%)
T2	555 (82.1%)
Considering dropping out of school T1	
No	575 (85%)
Yes	101 (15%)
Smoking T1	
No	545 (80.6%)
Yes	131 (19.4%)

**Table 2:** Descriptive characteristics of the participants. The Tromsø Study – Fit Futures

	All (*N* = 676)		Difference scores T2-T1 *Mean (SD)*
	T1 *Mean (SD)*	T2 *Mean (SD)*	
Age (years)	16.23 (0.91)	18.33 (0.91)	
HSCL-10	1.51 (0.53)	1.57 (0.60)	0.06 (0.52)
Steps per day / 1000	8.01 (3.61)	7.30 (2.98)	−0.712 (4.33)
MVPA (minutes per day)	44.66 (17.97)	36.46 (20.34)	−8.19 (25.33)
BMI (kg/m^2^)	22.27 (3.95)	23.19 (4.30)	
Hours of sleep	6.92 (2.26)	6.64 (2.41)	
Self-reported health (1–5)	3.92 (0.83)	3.89 (0.82)	
Unhealthy diet (times in a week)	2.51 (0.58)	2.36 (0.59)	
Healthy diet (times in a week)	2.62 (0.62)	2.66 (0.62)	
Social network (1–4)	3.30 (0.48)	3.23 (0.52)	

#### The relationship between change in physical activity and change in mental distress

Change in steps per valid day showed no statistically significant relationship with mental distress neither in the crude, partially or fully adjusted analyses (Table 3).

**Table 3:** The predictive effect of change in steps per valid day on change in mental distress from the Tromsø Study – Fit Futures 1 and 2

	B	95% CI	*p*
Crude model			
Change in steps per day/1000	−.005	−.021/.011	.538
Adjusted model Block 2^a^			
Change in steps per day/1000	−.005	−.021/.011	.571
Adjusted model Block 3^b^			
Change in steps per day/1000	−.004	−.019/.012	.640
Adjusted model Block 4^c^			
Change in steps per day/1000	−.003	−.018/.013	.751

^a^ Block 1: Model adjusted by demography: sex, age, socioeconomic status mother and father (T1), season of actigraphy measurement (T1 and T2)

^b^ Block 2: Model adjusted by variables in block 1 and social level variables: considering dropping out of school (T1), social network (T1) and change in social network (T2 –T1)

^c^ Block 3: Model adjusted by variables in block 1, block 2 and health and lifestyle related variables: chronic disease (T1), BMI (T1), sufficient sleep (T1), delayed sleep onset latency (T1), mean hours of sleep per night (T1), self-reported health (T1), change in self-reported health (T2-T1), junk food diet (T1), change in junk food diet (T2-T1), healthy diet (T1), change in healthy diet (T2-T1), chronic pain (T1) and smoking (T1)

Change in minutes in MVPA per valid day did not show any statistically significant association with change in mental distress (Table 4).

**Table 4:** The predictive effect of change in minutes in MVPA per valid day on change in mental distress from the Tromsø Study – Fit Futures 1 and 2

	B	95% CI	*P*
Crude model			
Change in minutes in MVPA per day	−.001	−.003/.001	.543
Adjusted model Block 2^a^			
Change in minutes in MVPA per day	−.001	−.003/.002	.577
Adjusted model Block 3^b^			
Change in minutes in MVPA per day	−.001	−.003/.002	.616
Adjusted model Block 4^c^			
Change in minutes in MVPA per day	.000	−.002/.002	.656

^a^ Block 1: Model adjusted by demography: sex, age, socioeconomic status mother and father (T1), season of actigraphy measurement (T1 and T2)

^b^ Block 2: Model adjusted by variables in block 1 and social level variables: considering dropping out of school (T1), social network (T1) and change in social network (T2 –T1)

^c^ Block 3: Model adjusted by variables in block 1, block 2 and health and lifestyle related variables: chronic disease (T1), BMI (T1), sufficient sleep (T1), delayed sleep onset latency (T1), mean hours of sleep per night (T1), self-reported health (T1), change in self-reported health (T2-T1), junk food diet (T1), change in junk food diet (T2-T1), healthy diet (T1), change in healthy diet (T2-T1), chronic pain (T1) and smoking (T1)

The interaction effects between change in steps per valid day or minutes in MVPA per valid day and sex were not statistically significant, nor were the interaction effects between change in steps per valid day or minutes in MVPA per valid day and baseline levels of mental distress (low, moderate and high baseline mental distress). Finally, the analysis of covariance of high negative to high positive change in steps per day on change in mental distress was not significant either.

## Discussion

When controlling for relevant covariates, the present study shows that change in steps per valid day and minutes in MVPA were unrelated to change in mental distress from the first to the third level of upper secondary school. This suggests that a change in physical activity is not related to change in mental distress among adolescents, regardless of the baseline levels of mental distress and levels of change in physical activity between baseline and follow-up.

The non-significant relationship between physical activity and mental distress contradicts several longitudinal studies based on self-reported physical activity [4, 21–24], but is in agreement with results from longitudinal studies based on objective measurements of physical activity [27, 28]. This discrepancy in results based on methodology may be crucial. In a study by Slootmaker et al. [51], adolescents on average reported having spent over 9 h more in moderate physical activity per week and almost 3 h more in vigorous activity per week than was assessed by accelerometer. Thus, the validity of self-reported physical activity among adolescents is questionable, and inferences made by analyzing data based on self-report may also be invalid. One can also suspect that depressed individuals tend to underreport positive behaviors, like physical activity, compared to more optimistic individuals, and in this way construct a negative, spurious relationship between mental health and physical activity. This is a weakness of self-report data that does not occur in accelerometer measurements. However, accelerometer measurements also have some limitations, and these are discussed in the section of methodological considerations below.

Compared to the present study, the follow-up period was shorter (1 year) in the longitudinal study of Van Dijk et al. [28] and longer (3 years) in the longitudinal study by Toseeb et al. [27]. Van Dijk and colleagues argued that the short follow-up period might have affected the results, as effects of changes in physical activity on mental health may take some time. Body composition changes are not immediate, neither are mastery and skill acquisition of sports or other kinds of physical activities. In our study, the mean number of months between T1 and T2 was 24.34, but for some of the participants the follow-up period was considerably shorter. Thus, the time between T1 and T2 may not have been sufficient for positive effects of physical activity to have occurred, and consequently not have had an impact on mental distress. However, as similar results were found in the study by Toseeb [27], with a follow-up period of 3 years, it suggests that the lack of significant effects is not due to short follow-up time. A limitation of our study as well as the study by Van Dijk et al. [28] and Toseeb et al. [27] is that the relationship was investigated over two time points only. Future studies should explore the relationship over multiple time points, in order to get a better understanding of the covariance between physical activity and mental distress.

The change in mean levels of physical activity in our study between baseline and follow-up was very small, as was the change in mean levels of mental distress. Thus, the lack of a significant effect of change in physical activity on change in mental distress could have been explained by a lack of significant change in activity between baseline and follow-up. The same could be true for the results of the Van Dijk et al. [28] study, as they also observed only a small change in physical activity and depressive symptoms over time. In order to see if the low mean change in activity disguised an effect among the adolescents who in fact did experience significant change between baseline and follow-up (a change in +/− 3000 steps), an analysis of covariance was performed. The effects were not significant. There is also a possibility that change in physical activity is not linearly related to change in mental distress, although at this point this is only speculation. However, several studies have shown that adolescents who are highly involved in (competitive) strenuous physical activity are at increased risk to suffer from symptoms of burnout and depression [52, 53]. Some individuals with high positive change in physical activity levels may have higher scores on mental distress, thus, counterbalancing the possible preventive effect of physical activity on mental distress.

The relatively large standard deviations of the change in steps, minutes in MVPA and mental distress (as shown in Table 2), suggest that there is variability in change over the 2 years between data collection. However, as discussed above, we do not know the timing at which the change occurred, or the duration of the change in physical activity levels. Recent changes in activity may not have lasted long enough to have been able to bring about change in mental health related factors. This means that more recent change may have watered down the effects of more long-lasting change in physical activity on mental distress.

As the effect of physical activity on mental health seems to be present in adulthood, but not in adolescence, this could indicate that physical activity serves another function for adolescents than for adults. A large part of the physical activity of adolescents is accrued through organized sports participation [54], but the adolescent period is also the period where dropout from organized sports mainly occurs [55]. Naturally, during the period before an individual decides to quit organized sports, exercising and being physically active may have negative connotations for the individual. A decline in physical activity between T1 and T2 for these individuals may thus have had a positive effect on mental health, and in that way cancelled out the negative effect on mental distress of a decline in physical activity between T1 and T2 for other participants.

There is also a possibility that other factors may play a more significant role in the association with mental distress than physical activity. It has been suggested that hormones that emerge in this period are partially to blame for the increased risk of affective disorders [56], but other risk factors may also have played a part. One study found that adolescents who participated in organized sports, showed lower odds for depressive symptoms than participants who reported that they kept fit independently (swimming, cycling or running) [15]. The current study fails to control for sport participation and physical activity performed in a social setting, and this is also true for other potentially influential factors, such as, self-esteem [57] and body image [58].

It is, however, necessary to point out that the majority of the previous studies on the relationship among adults have also been based on self-reporting as mentioned in the review by Mammen and Faulkner [59]. More research using objective measures of physical activity are needed both in adult and adolescent samples, in order to determine the degree to which physical activity may prevent the development of mental distress over time.

Some specific mental disorders often emerge in the adolescent years where physical activity is a key factor. Disordered eating or eating disorders, such as anorexia nervosa, are related to anxiety and depression [60] and typically develop in adolescence [61]. Disordered eating is common among adolescents (between 14 and 22% prevalence rates) [62], and one of the core features of disordered eating is excessive physical activity, in order to increase energy expenditure and lose weight. Exercise in young women with disordered eating has been found to be related to negative affect [63]. Disordered eating was not assessed in the present study, whereas the presence of any chronic disorders, such as eating disorders, were registered at T1, but not at T2. As we were unable to account for disordered eating and only to a small extent able to adjust for eating disorders, this may have affected our results. Future studies should include measures of disordered eating or motives for exercise in order to account for the variance explained by this factor.

It is also worth mentioning that intervention studies comparing the effects of physical activity interventions to control conditions have found physical activity to significantly reduce symptoms of depression among adolescents [64], which suggests that physical activity may be an effective component in treating existing depression. This may also suggest that the most mentally distressed individuals at T1 may profit more from a positive change in physical activity. However, the moderating effect of level of mental distress at T1 on the relationship between change in physical activity on change in mental distress was also non-significant. This means that change in physical activity was not related to change in mental distress even among the most highly distressed individuals at T1. Despite this result, we strongly recommend further explorations into how physical activity is related to change in mental distress among the individuals suffering from high levels of symptoms of anxiety and depression, as effectiveness and efficacy studies show that physical activity may benefit this group in terms of lower symptomatology [65].

### Methodological considerations

The major strength of this study is that physical activity was measured objectively and longitudinally in a relatively large sample, and that we were able to adjust for several potential confounders. Nonetheless, the results should be interpreted in light of certain methodological limitations.

Although objectively measured physical activity via accelerometer is considered more accurate and unbiased than self-reported physical activity, there are also validity issues concerning these methods that may have affected the results. Accelerometers have a weakness in the ability to reliably register different types of activities, such as cycling and weight lifting, because of its static position on the hip. In addition, the algorithms used to assess the physical activity levels are not fully explained by the cooperation behind the devices, and thus, the resulting physical activity levels are difficult to validate. It is also possible that the short period of wear time (7 days) was not representative for the person’s typical level of physical activity, as it may lead to over –or underestimation of the respondent’s normal levels of physical activity. There may have been issues both causing the levels of activity to increase and decrease, relative to normal levels. MVPA regressed towards the mean as its levels had a sharper decline for active participants in T1, compared to inactive participants (results not shown), and this regression may partially be explained by over – or underestimation of activity at either time point. However, research done on blinded and non-blinded groups show that the awareness of wearing an accelerometer has no effect on pattern of physical activity in adolescents [66]. We used uniaxial accelerometer measurements, and triaxial measurements may provide higher PA values, although not necessarily more accurate [67].

A relatively large amount of the data was imputed, due to a high percentage of missing values. This could potentially have introduced bias in the estimates. For multiple imputations to produce unbiased estimates, missing data must be missing at random. As we do not know the reason for why data is missing, there is always a chance that data is not missing at random. However, the degree to which the participants with missing data differ from the participants with valid data may provide an indication regarding a pattern in the reason for missingness. In our case, the respondents with missing data did not differ from the respondents with valid data. Although this does not guarantee that data is missing at random, it suggests a higher probability of it. Provided that data is missing at random, multiple imputation provides unbiased estimates, even with high proportions of missing values [68].

In addition, the results cannot be generalized to the Norwegian population of adolescents as a whole as the participants were selected from only two municipalities. Mental health was measured by self-reporting symptoms the last week prior to the study, and the period for the measurement may be too short to be able to measure the typical behavior. It is also possible that, in a sample where very few adolescents experience change in their mental health, it is difficult to find effects of change in activity levels. Finally, although the sample size is relatively large, the study may have benefitted from collecting data from an even larger sample. A larger sample could also have enabled the investigation of subgroups in which the relationship between physical activity and mental distress may differ.

## Conclusions

This study of adolescents from the general population found no significant longitudinal relationship between change in physical activity and change in mental distress over the period of upper-secondary school. These findings are in agreement with the two existing studies based on the same, sound methodology.

We recommend that future studies in the field investigate the relationship using objective measurements over multiple time points, but also include measures of other factors, such as motives for physical activity, context of activity, self-esteem and levels of mental distress, that may be important for gaining a better understanding of the possible relationship between physical activity and mental distress among adolescents.

## Data Availability

The dataset supporting the articles findings is available through application directed to the Tromsø Study by following the steps presented on their online page: https://en.uit.no/forskning/forskningsgrupper/sub?p_document_id=453582&sub_id=71247.

## Abbreviations

BMI: Body Mass Index
HSCL-10: Hopkins Symptom Checklist − 10
MVPA: Moderate to vigorous physical activity
